# Characteristics of Brazilian melanomas: real-world results before and after the introduction of new therapies

**DOI:** 10.1186/s13104-019-4336-7

**Published:** 2019-05-28

**Authors:** Letícia Maria Modes da Costa, Camila de Souza Crovador, Carlos Eduardo Barbosa de Carvalho, Vinicius de Lima Vazquez

**Affiliations:** 1 Department of Surgery, Melanoma, Sarcoma and Mesenchymal Tumors, Barretos Cancer Hospital, Antenor Duarte Villela, 1331, Barretos, SP 14784-400 Brazil; 2 Barretos School of Health Sciences, Dr. Paulo Prata, Medicine, Barretos, SP Brazil

**Keywords:** Melanoma, Skin neoplasms, Prognosis, Survival, BRAF inhibitor

## Abstract

**Objective:**

This study is a characterization of the treatment patterns and outcomes of a Brazilian melanoma cohort collected of 1848 patients enrolled between 1996 and 2015.

**Results:**

The superficial spreading subtype (35.1%) was the most prevalent, and the favoured anatomical location was the trunk (32.8%). The most common clinical stage was I (27.6%). The most frequent initial treatment was surgery (84.7%). Sentinel node biopsy was positive in 23.3% of cases. Chemotherapy was used to treat 298 patients (16.1%), immunotherapy for 67 (3.6%) and targeted therapy for 19 (1.0%). Distant recurrence was commonly observed (22.5%) and the mutation status of the *BRAF* gene was verified in 132 cases, with 42.4% positivity in this subset of patients. The melanoma specific actuarial 5-year survival for the cohort was 68.8%. There was a higher 5-year survival observed in metastatic melanoma patients who received immunotherapy and/or targeted therapy (34.2%) compared patients treated with just chemotherapy (20.0%). The survival analysis showed that sex, age, Breslow, clinical stage and distant recurrence were significant prognostic factors. This study provides a real-world description of how the introduction of new therapies such as immunotherapy and BRAF inhibitors is changing treatment strategies for melanoma in developing countries.

**Electronic supplementary material:**

The online version of this article (10.1186/s13104-019-4336-7) contains supplementary material, which is available to authorized users.

## Introduction

There were estimated to be 18.1 million new cases of cancer and 9.6 million deaths from cancer in the world in 2018. For melanoma, there were 287,723 new cases (1.6% of total cancer cases) and 60,712 deaths (0.6% of total deaths) [1]. Despite the low incidence of melanoma in the world, the high amount of sun exposure in Brazil means that there is a higher risk of this disease for fair-skinned people and for families and individuals with an elevated risk of skin cancer. There are expected to be 2720 new cases for men and 3340 new cases for women per 100,000 individuals in Brazil, with the highest proportion of melanomas being seen in the southern region of the country [2, 3].

Melanoma, the most aggressive of cutaneous neoplasms, is a tumor originating from melanocytes, which are the melanin-producing cells [4]. Surgery is the preferred treatment for localized melanoma and for melanoma with regional dissemination. Usually, the primary tumor is treated with wide local excision and presence of disease in regional lymph node is diagnosed trough sentinel node dissection. Lymphadenectomy is performed if sentinel node is metastatic or mostly in cases of macroscopic metastasis. Disseminated melanoma, may be treated surgically in selected cases. Cutaneous, subcutaneous and lymph node metastases from distal chains are associated with greater survival than visceral metastases and are also more frequently submitted for surgical resections [5].

Until recently patients with multi-metastatic disease would have been treated with systemic chemotherapy. Currently there are two new classes of drugs: targeted therapy against tumors with *BRAF* gene mutations and immunotherapy using immunological checkpoints inhibitors (anti CTLA-4 and anti-PD-1/PDL-1). These changes in treatment have profoundly impacted patient survival and have been incorporated into clinical practice even in the adjuvant setting for locally advanced disease [6–9].

In this study we sought to characterize Brazilian patients with melanoma according to demographic, clinical, histopathological, molecular and treatment data, and to analyze the associations of these aspects among themselves and with relapse and cancer—specific survival in an updated manner in the context of recent therapeutic changes. For this reason we used data from a single tertiary institution leader in the treatment of cancer in Brazil [10].

## Main text

### Methods

This is a retrospective study of patients seen at the Barretos Cancer Hospital (BCH), São Paulo State, Brazil during the period 1996 to 2015. All data were collected from medical records following the appropriate ethical guidelines. The study group does not include melanoma patients with incomplete clinical data or with follow-up times of less than 6 months from their first hospital visit. The variables compared comprised demographic; clinical/histological; treatment-related factors; molecular biomarker; and recurrence/survival.

The systemic treatment of the cohort was based on dacarbazine monotherapy as first-line and taxane/platine derivatives for second-line until 2012. From 2012 on, anti-BRAF monotherapy and anti-CTLA-4 were introduced. More recently combinations of anti-BRAF with anti-MEK and anti-PD-1 were iniciated as first-line options.

Melanoma-specific disease-free survival times were calculated from the date of primary tumor diagnosis, regional metastasis, and distant metastasis until the last event (clinical evaluation or death).

To identify associations between the demographic, clinical, histopathological and molecular characteristics of the population, the Fisher’s test or Chi square test was used and for continuous variables, the Anova test. Associations of variables to disease-free and cancer specific survival were assessed by the Kaplan–Meier method, with any differences being tested using the log-rank. Multivariate analyses were performed using the Cox regression model for significant variables using univariate analysis or they were considered fit in the multivariate model.

### Results

The study included 1848 patients that are detailed in Additional file 1: Table S1. The frequency of melanoma in the male and female was similar, the proportion of patients with white or light-coloured skin corresponded to 93.1%, and those with chronic sun exposure corresponded to 64.8% (Additional file 1: Table S1). Patients with black or other skin colour present lower limbs anatomical location than those with white skin (49.6 versus 25.1%, p < 0.001), as does the histological subtype of acral lentiginous (27.7 versus 6.8%, p < 0.001). A lower limb anatomical location was more prevalent in women than in men (32.7 versus 20.2%, p < 0.001), whereas for men the trunk was more frequently involved (37.2 versus 28.3%, p < 0.001). The superficial spreading histological subtype was the more common in women than men, but the nodular subtype was more common in men than women (29.6 versus 21.2, p = 0.003).

The mutation status of the *BRAF* gene was included in the investigation of patients with melanoma in more recent periods. Additional file 1: Table S1 shows that the examination was performed in 132 cases, with positivity in 56 (42.4%). Among the patients who obtained a positive result, 18 used anti-BRAF targeted therapy.

Follow-up duration ranged from 0 to 293.76 months [mean 47 (SD 44.1) median, 31 months]. Survival analysis was possible for 1514 patients. The mean survival for melanoma was 148 months (SD 6.6, median of 191.3). The estimated 5-year disease-specific survival (DSS) was 68.8%.

**Fig. 1:**
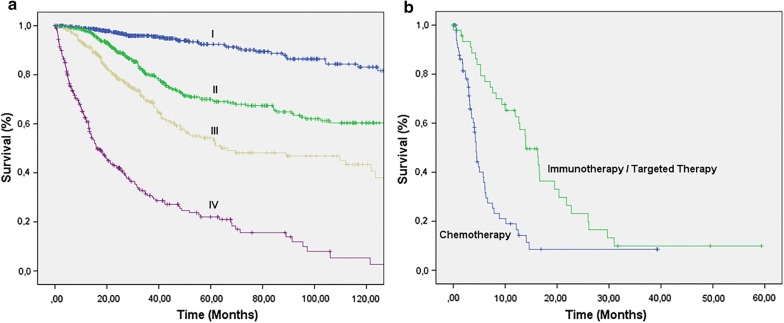
**a** Melanoma-specific survival according to clinical stage (p < 0.001) n = 1515 (Additional file 1: Table S4). **b** Stage IV melanoma-specific survival according to different therapies (p < 0.001). Immuno/targeted therapy n = 59. Only chemotherapy n = 227

Figure 1a and Additional file 1: Table S4 show the 5-year melanoma-specific survival by clinical stage. Our findings show that survival decreased as stage increased, with 92.4% survival for stage I, 69.5% for II, 53.5% for III and just 22.0% for IV. Comparative analyses of survival in metastatic patients were performed based on the various treatment received during the study period. The 5-year cancer-specific survival of patients treated with chemotherapy alone was 20% but was 34.2% in patients who also underwent targeted or immunotherapy (p = 0.015) (Fig. 1b and Additional file 1: Table S4). Considering the time of initiation of sistemic the median survival was 13.96 months in those treated with targeted therapy or immunotherapy and 4.401 in those who received just chemotherapy (p < 0.001) (Additional file 1: Table S4 and Fig. 1b).

The supplementary material has information about the treatment (Additional file 1: Table S2) and univariate analysis of melanoma-specific survival (Additional file 1: Table S3). Additional file 1: Table S5 shows the positive sentinel node biopsy according to the Breslow thickness.

According to clinical stage and recurrence status, all TNM variables, presence of locoregional or distant recurrence, and serum lactate dehydrogenase (LDH) levels were associated with prognosis (Additional file 1: Table S4). The multivariate analysis showed that age, sex, Breslow depth, TNM stage, lactate dehydrogenase levels at diagnosis and distant recurrence were associated with prognosis (Table 1).

**Table 1:** Multivariate analysis of melanoma-specific survival

Variable	Category	*n*	HR	95% CI (HR)	p value
Sex	Male	327	1.000	–	–
	Female	360	1.445	1.031; 2.026	0.032
Age	–	687	1.016	1.004; 1.028	0.009
Mitosis	–	687	1.007	0.977; 1.037	0.657
Breslow	–	687	1.047	1.005; 1.091	0.028
Clark	–	687	0.999	0.797; 1.253	0.993
Ulceration	Absent	382	1.000	–	–
	Present	305	1.002	0.678; 1.481	0.991
Clinical stage	I	257	1.000	–	–
	II	234	0.176	0.037; 0.847	0.030
	III	141	0.486	0.111; 2.130	0.338
	IV	41	0.802	0.181; 3.547	0.771
	X	14	2.117	0.463; 9.678	0.333
Locoregional recurrence	No	577	1.000	–	–
	Yes	110	0.851	0.589; 1.228	0.387
Distant recurrence	No	539	1.000	–	–
	Yes	148	2.285	1.887; 2.766	< 0.001

### Discussion

There have been many cohort studies describing the clinical characteristics of patients with melanoma and their prognosis, but in Brazil, these data are scarce and a more comprehensive approach is needed to better understand the impact of new therapies.

The mean age of diagnosis in our cohort was within the sixth decade of life, which is similar to the world data. The frequencies of melanoma in males and females were also similar to other studies in Brazil and in the world that show there are no predisposing risk for either gender [11].

The prognosis is considered favourable if the cancer is detected at an early stage [12–15]. In recent years there has been a significant improvement in the survival of patients due to early detection. In developed countries, the estimated 5-year survival rate is 73%; in developing countries, the survival rate drops to 56%, and the estimated global average is 69%. In Brazil, in work we published previously, this figure was 67.6% [16]. This study, which contains updated information from this same hospital, had a 5-year cancer-specific survival rate of 68.8%.

The 5-year survival rate of melanoma patients at the localized stage is 98.5% versus 19.9% in metastatic disease. This can also be observed in 5-years survival according to clinical stage, in which stage I was 92.4%, II 69.5%, III 53.5% and IV 22%, according to the literature [16, 17]. The fact that initial stages survival rates were lower than previously described can be explained because of the exclusion of patients with melanoma in situ (stage 0) in this analysis [18].

The presence of intratumoural and peritumoural lymphocyte infiltration, a known factor related to prognosis, did not affect the survival rates in this study. Other factors, such as sex, anatomic location, histological subtype, vascular and/or lymphatic infiltration, perineural invasion presence of regression, microscopic satellitosis, mutation in BRAF gene, pT, pN, pM, serum lactate dehydrogenase level, were associated with prognosis and lower survival rates in the univariate analysis. However, these variables could not be tested in the multivariate model because of the relatively small number of patients who remained in the model (687). The variables that remained in the multivariate model were Clark classification, Breslow depth, mitotic index, presence of ulceration, clinical stage, distant recurrence, and locoregional recurrence, whereas the clinical stage, age, and recurrence the distance were the main factors related to prognosis. The variables that were not associated with literature findings are probably due to the small sample in our study, compared to the 17,600 cases that were used to validate the UICC/AJCC staging system [19].

In the present study, comparing the effects of the *BRAF* gene mutation test, the 5-year survival of those with a positive test finding was significantly higher than those with a negative test (31.4 versus 18.5) by univariate analysis, but significance was not sustained in multivariate analysis. Patient tumours that were negative for the BRAF mutation tended to be younger than those that were positive. This difference can explain the survival advantage, since younger patients typically present with a higher survival in keeping with the literature [20].

The treatment modalities used in this study followed the international trends with the vast majority of patients having surgery as the first treatment, and only 15.3% of the patients were not submitted for surgery. The systemic therapies used at our institution include chemotherapy, immunotherapy, and targeted therapy. The latter were indicated only recently for use in stage IV or recurrence or inoperable stage III disease. Dacarbazine was used as first-line chemotherapy, the second-line was carboplatin and paclitaxel. Immunotherapy was most commonly performed with anti CTLA-4 and anti-PD-1. The most commonly used targeted therapy was anti-BRAF and the combination of anti-BRAF/MEK drugs. Radiotherapy was indicated primarily to attenuate metastases and, in selected cases, as adjuvant after lymphadenectomy [21].

The creation of anti-CTLA-4 and anti-PD-1 antibodies has been shown to be successful in studies and clinical practice as a generic therapeutical tool (non-targeted), with impressive survival up to 50% in metastatic patients [8, 22, 23]. A considerable proportion of melanomas (about 40–50%) and also nevitic lesions present activating mutations in the BRAF oncogene (over 90% V600E) [24]. The discovery and use of molecules that block the protein resulting from the mutation has been successfully employed in the clinical context since 2011, with high response rates and survival gain in phase III studies [6, 25, 26]. The best survival observed in patients who used immunotherapy and/or targeted therapy when compared to patients who only used chemotherapy demonstrates the significant progress with the introduction of these new therapies (Fig. 1b).

Locoregional recurrence had no association with survival in the multivariate analysis of this study. Distant recurrence was strongly related to prognosis, with lower survival rates, which indirectly shows the importance of choosing the best treatment in order to avoid recurrence.

### Conclusions

The melanoma-specific survival of the Brazilian population is less than the global average. However, our analysis elucidates the impact of the introduction of new therapies on the survival of the patients, confirming continuing progress in the treatment of melanoma. Although we demonstrate advances in survival of metastatic patients, effective strategies for earlier diagnoses are still necessary to achieve similar results of developed countries.

## Limitations

This retrospective study of a single institution has several limitations, such as patients with incomplete data and selection bias due to changing patterns of treatment. However, our ongoing findings continue to be important for multi-institutional studies to obtain a better understanding of possible socioeconomic disparities in different parts of Brazil that may affect access to treatment. Collectively our analyses are making it possible to design realistic strategies for early detection and treatment in developing countries.

## Additional file

**Additional file 1: Table S1.** Demographical, histological and clinical characteristics of 1848 melanoma patients. **Table S2.** Treatment characteristics of 1848 melanoma Patients. **Table S3.** Melanoma-specific survival (DSS) according to demographic, clinical and histological characteristics. **Table S4.** Melanoma-specific survival (DSS) according to clinical stage, recurrence status and therapy. **Table S5.** Tumour thickness in patients submitted to sentinel node biopsy.

## Data Availability

The datasets of this study can be made available on request forwarded to the corresponding author.

## Abbreviations

AJCC: American Joint Committee on Cancer
BCH: Barretos Cancer Hospital
DSS: disease-specific survival
HR: hazard ratio
LDH: lactate dehydrogenase
SD: standard deviation
SNB: sentinel node biopsy
UICC: International Union Against Cancer
