# Supplementary material for: Characteristics of Brazilian melanomas: real-world results before and after the introduction of new therapies
Source: BMC Res Notes. 2019 May 28;12:296. doi: 10.1186/s13104-019-4336-7 (PMC6537357; doi:10.1186/s13104-019-4336-7)
Supplement: Supplementary file 1 — Additional file 1: Table S1. Demographical, histological and clinical characteristics of 1848 melanoma patients. Table S2. Treatment characteristics of 1848 melanoma Patients. Table S3. Melanoma-specific survival (DSS) according to demographic, clinical and histological characteristics. Table S4. Melanoma-specific survival (DSS) according to clinical stage, recurrence status and therapy. Table S5. Tumour thickness in patients submitted to sentinel node biopsy. [file 13104_2019_4336_MOESM1_ESM.pdf]

Table S1 Demographical, histological and clinical characteristics of 1848 melanoma patients

Patient characteristics	Range	Mean
Age	1-100	56.69
**Patient characteristics**	**Number**	**%**
**Sex**		
Male	912	49.40%
Female	935	50.60%
**Skin colour**		
White	1677	93.10%
Other	124	6.90%
**Sun Exposure**		
Chronic	344	64.80%
Intermittent	33	6.2%
None	154	29%
**Anatomical location (primary tumour)**		
Trunk	606	32.8
Lower extremities	491	26.6
Head and neck	366	19.8
Upper extremities	251	13.6
Unknown	61	3.3
Others	22	1.2
Not available	51	2.8
**Histological subtype**		

Superficial spreading	649	35.1
Nodular	394	21.3
Acral lentiginous	130	7.0
Lentigo maligna melanoma	66	3.6
Other/not classified	320	17.4
Not available	289	15.6
**Tumour depth (Breslow)**		
Up to 1.0 mm	468	25.3
1.1 to 2.0 mm	270	14.6
2.1 to 4.0 mm	239	12.9
More than 4.0 mm	357	19.3
Not available	514	27.8
**Clark classification**		
I	52	2.8
II	180	9.7
III	427	23.1
IV	523	28.3
V	195	10.6
Not available	471	25.5
**Ulceration**		
Present	504	27.3
Absent	678	36.7
Not available	666	36.0
**Mitotic index**		
Up to 1 mitosis/mm2	249	13.5

More than 1 mitosis/mm2	524	28.4
Not available	1075	58.2
**Mutation Test of the BRAF Gene**		
No	564	81.0
Yes	132	19.0
Negative	74	56.1
Positive	56	42.4
Inconclusive	2	1.5
Anti-BRAF Therapy	18	32.1
**Clinical stage (AJCC)**		
0	162	8.8
I	510	27.6
II	422	22.8
III	317	17.2
IV	270	14.6
Not available	167	9.0

Table S2 Treatment characteristics of 1848 melanoma Patients

Treatment	Number	%
**Surgery (primary tumour)**		
Not operated	283	15.3
Primary closure	570	30.8
Local surgery	578	31.3

Local surgery and reconstruction (graft or flap)	369	19.9
Amputation	92	5.0
Second intention healing	35	1.9
**Sentinel node biopsy**		
Positive	148	23.3
Negative	486	76.7
**Adjuvant therapy**		
No	1673	90.5
Radiation	47	2.5
Systemic	86	4.6
Not Available	58	3.1
**Systemic therapy**		
No	1068	57.8
Yes	313	16.9
Not available	467	25.3
Chemotherapy	298	16.1
Immunotherapy	67	3.6
Targeted Therapy	19	1.0
**Radiation therapy**		
No	1120	60.6
Yes	242	13.1
Not available	486	26.3

Table S3 Melanoma-specific survival (DSS) according to demographic, clinical and histological characteristics

**Variable**	**Categories**	***N***	**5-year DSS (%)**	**P**
Sex	Male	766	57.1	<0.001
	Female	748	74.0	
Skin colour	White	1375	66.7	0.018
	Other	103	57.2	
Anatomical location	Upper limbs	191	73.3	<0.001
	Trunk	513	72.6	
	Head and neck	267	64.4	
	Lower limbs	427	63.0	
	Unknown	58	31.0	
Clark	I	7	75.0	<0.001
	II	164	95.2	
	III	397	81.5	
	IV	489	69.5	
	V	186	38.9	
Histological subtype	Acral lentiginous	122	47.7	<0.001
	Nodular	362	60.7	
	Superficial Spreading	590	86.2	
	Lentigo maligna	47	83.5	
	Not classified/other	147	74.9	

Mitosis/mm2	0-1	231	82.1	<0.001
	>1	496	63.4	
Intratumoural lymphocyte infiltration	Yes	527	70.4	0.884
	No	362	70.8	
Peritumoural lymphocyte infiltration	Yes	540	72.7	0.502
	No	183	71.6	
Vascular and lymphatic infiltration	Yes	56	43.4	<0.001
	No	741	72.5	
Perineural invasion	Yes	38	47.4	<0.001
	No	722	72.6	
Ulceration	Present	472	54.4	<0.001
	Absent	601	83.3	
Regression	Yes	117	57.1	<0.001
	No	772	71.0	
Microscopic satellitosis	Yes	64	46.8	<0.001
	No	768	74.7	

Mutation Test of the BRAF Gene	Positive	56	31.4	<0.001
	Negative	74	18.5	

Table S4 Melanoma-specific survival (DSS) according to clinical stage, recurrence status and therapy

**Variable**	**Categories**	***N***	**5-year DSS (%)**	**p**
T	T1	383	91.6	<0.001
	T2	270	80.8	
	T3	264	65.0	
	T4	367	49.7	
N	N0	972	80.7	<0.001
	N1	138	52.1	
	N2	99	46.2	
	N3	120	34.9	
M	M0	1245	74.6	<0.001
	M1	267	21.7	
Clinical stage (TNM)	I	508	92.4	<0.001
	II	421	69.5	
	III	317	53.5	
	IV	269	22.0	
Distant recurrence	Absent	1179	80.2	<0.001
	Visceral	252	22.4	

	Non-visceral	84	42.2	
Locoregional recurrence	Yes	246	36.3	<0.001
	No	1269	75.1	
Serum lactate dehydrogenase	≤480 IU/L	934	74.6	<0.001
	>480 IU/L	213	46.6	
Therapy	Immunotherapy/ Targeted Therapy	59	34.2	0.015
	Only Chemotherapy	227	20.0	

Table S5 Tumour thickness in patients submitted to sentinel node biopsy

**Tumour thickness (pT)**	**Total**	**Positive**	**Negative**
pT1, n (%)	80 (12.62)	9 (11.25)	71 (88.75)
pT2, n (%)	185 (29.18)	29 (15.67)	156 (84.32)
pT3, n (%)	127 (20.03)	44 (34.64)	83 (65.35)
pT4, n (%)	167 (26.34)	56 (33.53)	111 (66.47)
Not available, n (%)	75 (11.83)	10 (13.33)	65 (86.66)
Thickness in mm, mean (SD)	3.26	4.81	2.76
